# Angiotensin II‐induced renal angiotensinogen formation is enhanced in mice lacking tumor necrosis factor‐alpha type 1 receptor

**DOI:** 10.14814/phy2.14990

**Published:** 2021-08-24

**Authors:** Dewan S. A. Majid, Eamonn Mahaffey, Alexander Castillo, Minolfa C. Prieto, L. Gabriel Navar

**Affiliations:** ^1^ Department of Physiology Hypertension & Renal Center of Excellence Tulane University School of Medicine New Orleans Louisiana USA

**Keywords:** angiotensin II, angiotensinogen, High salt intake, renal injury, TNF‐α receptors

## Abstract

In hypertension induced by angiotensin II (AngII) administration with high salt (HS) intake, intrarenal angiotensinogen (AGT) and tumor necrosis factor‐alpha (TNF‐α) levels increase. However, TNF‐α has been shown to suppress AGT formation in cultured renal proximal tubular cells. We examined the hypothesis that elevated AngII levels during HS intake reduces TNF‐α receptor type 1 (TNFR1) activity in the kidneys, thus facilitating increased intrarenal AGT formation. The responses to HS diet (4% NaCl) with chronic infusion of AngII (25 ng/min) via implanted minipump for 4 weeks were assessed in wild‐type (WT) and knockout (KO) mice lacking TNFR1 or TNFR2 receptors. Blood pressure was measured by tail‐cuff plethysmography, and 24‐h urine samples were collected using metabolic cages prior to start (0 day) and at the end of 2nd and 4th week periods. The urinary excretion rate of AGT (uAGT; marker for intrarenal AGT) was measured using ELISA. HS +AngII treatment for 4 weeks increased mean arterial pressure (MAP) in all strains of mice. However, the increase in MAP in TNFR1KO (77 ± 2 to 115 ± 3 mmHg; *n* = 7) was significantly greater (*p *< 0.01) than in WT (76 ± 1 to 102 ± 2 mmHg; *n* = 7) or in TNFR2KO (78 ± 2 to 99 ± 5 mmHg; *n* = 6). The increase in uAGT at 4th week was also greater (*p *< 0.05) in TNFR1KO mice (6 ± 2 to 167 ± 75 ng/24 h) than that in WT (6 ± 3 to 46 ± 16 ng/24 h) or in TNFR2KO mice (8 ± 7 to 65 ± 44 ng/24 h). The results indicate that TNFR1 exerts a protective role by mitigating intrarenal AGT formation induced by elevated AngII and HS intake.

## Graphical abstract

We examined the hypothesis that elevated AngII levels during HS intake reduce TNF‐α receptor type 1 (TNFR1) activity in the kidneys, thus facilitating increased intrarenal AGT formation. The responses to HS diet (4% NaCl) with chronic infusion of AngII (25 ng/min) via implanted minipump for 4 weeks were assessed in wild‐type (WT) and knockout (KO) mice lacking TNFR1 and TNFR2 receptors. HS +AngII treatment for 4 weeks increased mean blood pressure (MBP) in all strains of mice. However, the increase in MBP in TNFR1KO was greater than that in WT or in TNFR2KO. The increase in urinary angiotensinogen excretion (UAGT) at the 4th week was also greater in TNFR1KO mice than that in WT or in TNFR2KO mice. The results indicate that TNFR1 activity exerts a protective role by mitigating intrarenal AGT formation induced by elevated AngII and HS intake.

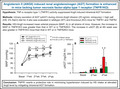

## 1 INTRODUCTION

Tumor necrosis factor‐alpha (TNF‐α) is a pro‐inflammatory cytokine that has a wide range of immune functions related to lymphoid development and inflammation, as well as pro‐apoptotic effects that protect against tumor proliferation (Afsar et al., 2018; Harrison et al., 2011; Majid, Prieto, & Navar, 2015; Mattson, 2014; Mehaffey & Majid, 2017). TNF‐α is predominantly produced by macrophages but can also be secreted in limited quantities by T and B lymphocytes, natural killer cells, endothelial and muscle cells, fibroblasts, and osteoclasts (Hao et al., 2020; Harrison et al., 2011; Mattson, 2014; Schiffrin, 2013). This cytokine is recognized to have important roles in the regulation of the cardiovascular system (Harrison et al., 2011; Mehaffey & Majid, 2017; Rodríguez‐Iturbe et al., 2014; Urschel & Cicha, 2015). In the kidneys, tubular epithelial and mesangial cells produce this cytokine in response to inflammation and tissue injury (Abdullah et al., 2006; Battula et al., 2011; Mattson, 2014; Muller et al., 2002; Ruiz‐Ortega et al., 2002). Under normal conditions, TNF‐α is undetectable in plasma, but its level becomes elevated in plasma and renal tissues of rodent models of hypertension induced by the chronic administration of angiotensin II (AngII; Castillo et al., 2018; Guzik et al., 2007; Lara et al., 2012; Sriramula et al., 2008), nitric oxide (NO) inhibitor agents (Shahid et al., 2008; Singh et al., 2014; Whiting et al., 2013), DOCA salt (Elmarakby et al., 2008), and also in Dahl salt‐sensitive rats (Huang et al., 2016).

In animal studies, chronic high salt (HS) intake causes no or minimal change in systemic blood pressure, but it exaggerates the hypertensive response induced by the chronic inhibition of NO (Kopkan et al., 2010) and in elevated AngII (Lara et al., 2012) conditions. An enhancement in angiotensinogen (AGT) generation by the kidneys is associated with the progression of renal injury in hypertension and in chronic kidney diseases (CKD) induced by HS intake (Kobori et al., 2003, 2007, 2009; Lara et al., 2012; Majid & Kopkan, 2007; Majid, Prieto, & Navar, 2015). Renal proximal tubule‐specific AGT overexpression and secretion increase intratubular Ang II levels and enhances the development of salt‐sensitive hypertension (Kobori et al., 2007). Although HS intake alone does not affect the renal generation of AGT, it augments the increases in intrarenal AGT production induced by chronic AngII treatment (Lara et al., 2012). The reason for such an exaggerated increase in AGT in HS +AngII‐treated conditions is not clearly understood.

Ang II stimulates both TNF‐α (Ruiz‐Ortega et al., 2002) and intrarenal AGT formation (Kobori et al., 2007; Lara et al., 2012). However, the link between TNF‐α and AGT formation in the kidneys remains poorly understood. In studies have shown that TNF‐α suppressed AGT production in cultured human proximal tubule cells (Satou et al., 2010). TNF‐α exerts its biological responses via bindings with two cell surface receptors, TNF‐α receptor type 1 (TNFR1) and type 2 (TNFR2) which are differentially expressed and regulated in the kidneys (Castillo et al., 2012). Although the specific roles of these receptors in various conditions have not been fully delineated, TNF‐α binding of TNFR1 and TNFR2 elicit distinct signaling pathways, which result in cellular responses that can promote tissue injury on the one hand, but exerts protective, beneficial responses on the other hand (Majid, Prieto, & Navar, 2015; Mehaffey & Majid, 2017). Chronic Ang II infusion in TNFR1 knockout (KO) mice enhances the BP responses reflecting a protective role of TNFR1 during Ang II treatment (Chen et al., 2010). TNFR1 also mediates TNF‐α‐induced natriuretic responses in mice (Castillo et al., 2012; Majid, Prieto, & Navar, 2015; Shahid et al., 2008) indicating that TNFR1 would help to minimize salt retention during HS intake and AngII treatment. In contrast, the renal fibrotic lesions induced by chronic HS and AngII infusions were attenuated in TNFR2KO, indicating that TNFR2 exerts a contributory role in inducing renal injury (Singh et al., 2013). AngII treatment for a short‐term period of about 3 days downregulates TNFR1 and upregulates TNFR2 in the renal medulla (Ruiz‐Ortega et al., 2002). Preliminary experiments also reveal that HS intake for 4 weeks in mice enhances TNFR1 protein expression, while HS coupled with AngII treatment reduces TNFR1 expression and increases TNFR2 expression in the renal tissues (Majid, Prieto, & Castillo, 2015). Thus, these findings strongly suggest that the differential activity of TNF‐α receptors occurs during chronic HS intake and AngII treatment.

The present study examined the hypothesis that chronic elevation in AngII levels coupled with HS intake reduces TNFR1 activity but enhances TNFR2 activity that induces cellular responses causing increased formation of intrarenal AGT leading to an exaggerated hypertensive condition. We assessed the renal and systemic responses to chronic treatment with AngII and HS diet for 4 weeks in KO mice lacking the genes for TNFR1 (TNFR1KO) or TNFR2 (TNFR2KO) and compared these responses to those in wild‐type (WT) mice.

## 2 MATERIALS AND METHODS

The experiments were performed using the protocol in accordance with the established guidelines and practices approved by the Tulane University Animal Care and Use Committee. Male mice in which the TNF‐α receptors TNFR1 (B6.129‐Tnfrsf1atm1Mak/J; stock no. 002818) and TNFR2 (B6.129S2‐Tnfrsf1btm1Mwm/J; stock no. 002620) had been genetically knocked out and their background wild‐type (WT; C57BL/6; stock no. 000664) mice were used in these experiments (Jackson Laboratories). The knockout mice (TNFR1KO and TNFR2KO) as well as WT mice were housed in a temperature‐ and light‐controlled room and allowed free access to the standard diet (Ralston‐Purina) and water. The mice (8–9 weeks of age; ∼25 g body wt) were kept in the facility for ∼10 days before the start of each experimental protocol.

### 2.1 Blood pressure monitoring in conscious mice

Blood pressure was measured using a tail‐cuff plethysmograph (Visitech Systems). This plethysmograph allows the recordings of systolic and diastolic blood pressures which allows the calculation of mean arterial pressure (MAP) using its Analysis Software. Mice were trained for tail‐cuff blood pressure measurements 1 week before starting the experiments. Blood pressure measurements were performed at the same time of the day (11 AM to 1 PM) to standardize for the influence of the circadian cycle on blood pressure. The average blood pressure was obtained by calculating the average readings of 10 measurements for a single trial. Blood pressure was measured one day before the start of minipump implantation (0 day, considered as baseline) and then on days 7, 14, 21, and 28 of the 4‐week experimental period.

### 2.2 Chronic AngII administration by osmotic minipumps

Chronic infusion of AngII at a rate of 25 ng/min for a 4‐week period was given to all the mice using osmotic mini‐pumps (Alzet, model 1002D; Durect). During AngII infusion, the mice were given an HS diet (4% NaCl; Harlan‐Teklad) and allowed to drink freely from the water bottles attached to the cages. For the implantation of the osmotic mini‐pump, mice were anesthetized for a brief period using 2% isoflurane. The minipump containing AngII dissolved in 0.9% saline was implanted subcutaneously under the scapula through an incision in the mid‐scapular region under sterile condition. The antibiotic tetracycline (500 mg/L) was given in the drinking water one day before the surgery and continued for the 4‐week duration of the experimental period.

### 2.3 Urine collection in conscious mice

Twenty‐four‐hour (h) urine samples were collected from conscious mice using metabolic cages on the day before the start of the treatment to establish basal excretory parameters and then at the end of 2nd and 4th week periods of the experiment. Animals were housed individually in metabolic cages and urine was collected for 24 h into sterile tubes. Maximum precaution was taken to avoid the contamination of urine with chow food debris by covering the urine collection tubes with cling film. Urine volumes were determined from each urine collection, and samples were centrifuged (3,000 rpm/5 min; 4℃) and preserved for analysis. Food and water intake along with the body weight of each mouse was measured during the experimental period. Urinary concentrations of sodium and potassium were assessed by flame photometry. Urinary AGT excretion was quantified as an index of intrarenal production of AGT using ELISA. On day 28, all the animals were euthanized under anesthesia with inactin (Sigma‐Aldrich, 150 µg/g of body weight) and blood samples were collected by carotid artery cannulation. After exsanguination, the kidneys were collected and kept in formalin for the evaluation of renal injury.

### 2.4 Enzyme‐linked immunosorbent assay for the urinary AGT level

Urinary AGT (uAGT) concentrations in urine samples were measured by enzyme‐linked immunosorbent assay (ELISA) using a mouse AGT measuring kit (IBL‐America). The detection levels of AGT in the kits (standard curve range) are 0.31–20 ng/ml.

### 2.5 Evaluation of renal tissue injury

Formalin‐fixed paraffin‐embedded kidney sections were used for analyzing glomerulosclerosis using Periodic‐Acid‐Schiff (PAS) staining (Sigma Diagnostic; Fisher Scientific, Cat No. SD395B), and for the assessment of interstitial fibrosis using Artisan Gomori's Blue Trichrome staining (Agilent Technologies, Inc; AR167), as indicated by the manufactures. The extent of the interstitial fibrotic‐positive area was evaluated quantitatively using computerized image analysis, as previously described (Lara et al., 2012).

#### 2.5.1 Estimation of glomerulosclerosis

The extent of glomerulosclerosis was evaluated quantitatively in automatic images captured of each glomerulus visualized with a 40X magnification oil objective and PAS‐staining of renal sections. Mesangial expansion was quantified in 20 glomeruli per kidney section and then dividing the PAS‐positive stained area by the total area of the glomerulus. Values were expressed as means ± *SE* of the 20 measurements in percentage values, as previously described (Lara et al., 2012). Slides were photographed using a Nikon Eclipse 50i microscope equipped with a Nikon DS Camera Head (DS Fi1) and DS camera control unit (DSU2). The percentage area covered by sclerosis in the glomeruli in each field was analyzed using the NIS‐Elements software (version 3.0) image analyzer in a blinded manner to avoid bias.

#### 2.5.2 Estimation of interstitial fibrosis

The extent of interstitial collagen‐positive area (fibrosis) was quantitatively evaluated by an automatic image analysis of renal section occupied by interstitial tissue blue staining positively for collagen deposition as described previously (Lara et al., 2012). Briefly, formalin‐fixed paraffin‐embedded renal sections were sequentially processed in Bouin's solution, Hematoxylin A and B, Gomori's blue trichrome staining, and 0.5% acetic acid, as described by the manufacturers. Slides were photographed as described above and visualized with a 40X magnification of renal sections. The percentage area covered by blue staining represents collagen deposition in each field and was analyzed using the Nikon 50i microscope and the NIS‐Elements (AR version 3.0) software image analyzer in a blinded manner to avoid bias. Values of the percentage data obtained for each of the 20 captured analyzed images were averaged to obtain the percentage area of fibrosis for the entire slide.

### 2.6 Statistical analysis

Results were expressed as mean ± *SEM*. All excretory values were normalized as units per gram of kidney weight. Statistical analysis of the values within groups was conducted using the repeated measures ANOVA. Comparison of the responses (which are the differences in basal and treatment period values) in TNFR1KO or TNFR2KO groups with that in WT group were compared using One Way ANOVA analysis. Differences are considered significant at *p* < 0.05.

## 3 RESULTS

### 3.1 Responses to ANG II + HS treatment in TNFR1KO and TNFR2KO mice

#### 3.1.1 Blood pressure responses

**FIGURE 1:**
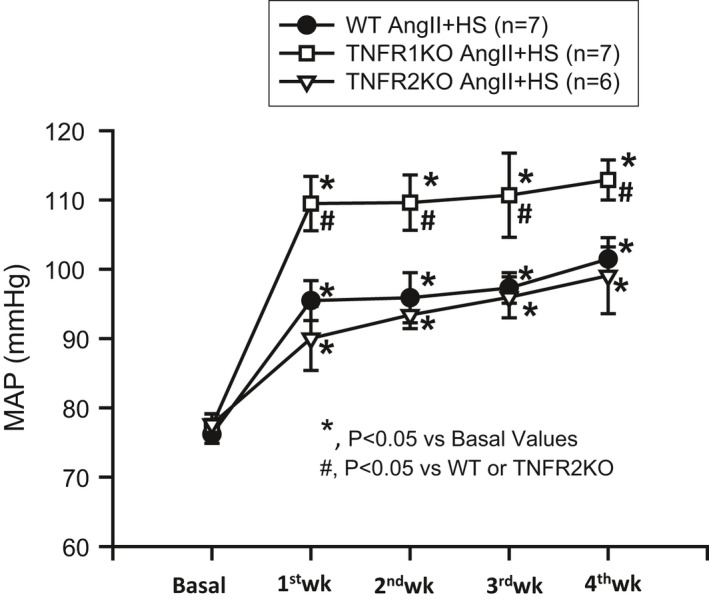
Mean arterial pressure (MAP) responses to chronic HS (4% NaCl in diet) intake + AngII (25 ng/min) treatment for 4 weeks. AngII +HS intake increases MAP in all strains of mice. However, TNFR1KO mice had significantly greater increases in MAP compared to wild‐type (WT) or TNFR2KO mice. No significant changes were seen in the MAP of TNFR2KO compared to WT

The basal mean arterial blood pressure (MAP) was the same in the three groups of mice (WT, TNFR1KO, and TNFR2KO) as demonstrated in Figure 1. Chronic administration of AngII with HS intake for 4 weeks increased MAP in all groups; however, the increase in MAP was greater in TNFR1KO mice compared to that in WT mice, while the MAP in TNFR2KO was not different from WT. The mean increases in MAP at the end of the 4‐week period were as follows: WT, 76 ± 1 to 102 ± 2 mmHg; TNFR1KO, 77 ± 2 to 115 ± 3 mmHg; TNFR2KO, 78 ± 2 to 99 ± 5 mmHg.

#### 3.1.2 Renal excretory responses

In Table 1, the values for daily water intake (WI), urine output (V), sodium (U_Na_V), and potassium excretion rate (UKV) are given which were deduced from metabolic cage collection of 24‐hour (h) samples in all groups of mice. AngII administration with HS intake for 4 weeks resulted in significant increases in daily WI, V, and U_Na_V but not in UKV. However, there were no significant differences in these responses among the groups. The mean calculated volume retention (differences between water intake and urine output) values by each group on the day of the treatment period (TNFR1KO, 4.89 ± 0.86 ml/24 h; TNFR2KO, 3.50 ± 1.01 ml/24 h; WT, 3.84 ± 1.05 ml/24 h) were also not significantly different among the groups. There were no significant differences in food intake as well as in the body weight of these mice among the different groups (data not included). All three groups showed similar increases in U_Na_V. However, UKV did not change significantly during the experimental period.

**TABLE 1:** Renal excretory responses to chronic HS (4% NaCl in diet) intake + AngII (25 ng/min) treatment for 4 weeks

Mice strains	WI (ml/24 h)			V (ml/24 h)			U_Na_V (mM/24 h)			UKV (mM/24 h)		
	Basal	2 week	4 week	Basal	2 week	4 week	Basal	2 week	4 week	Basal	2 week	4 week
WT (*n* = 7)	1.4 ± 0.3	7.8 ± 1.6*	11.1 ± 2.1*	0.9 ± 0.1	5.5 ± 1.0*	6.9 ± 0.9*	118 ± 13	847 ± 118*	883 ± 121*	262 ± 32	251 ± 24	287 ± 12
TNFR1KO (*n* = 7)	2.8 ± 0.2	4.9 ± 1.5*	13.8 ± 1.9*	0.9 ± 0.2	3.8 ± 0.9*	7.9 ± 0.8*	96 ± 14	706 ± 133*	942 ± 86*	248 ± 44	234 ± 32	230 ± 13
TNFR2KO (*n* = 6)	2.1 ± 0.3	4.6 ± 1.3*	11.4 ± 1.0*	0.9 ± 0.1	3.3 ± 1.1*	5.3 ± 0.8*	104 ± 13	681 ± 116*	732 ± 169*	235 ± 32	218 ± 39	235 ± 32

Note

The mean values of water intake (WI), urine output (V), sodium (U_Na_V), and potassium (UKV) excretion for 24 h metabolic cage collection samples during chronic HS (4% NaCl in diet) intake + AngII (25 ng/min) treatment for 4 weeks in different groups of mice. *p < 0.05 versus Basal values. Although the administration of ANGII and HS intake increased WI, V, and U_Na_V (but not UKV) in all three groups of mice, no significant differences were observed among groups in these increases in the urinary parameters.

#### 3.1.3 Urinary AGT responses

**FIGURE 2:**
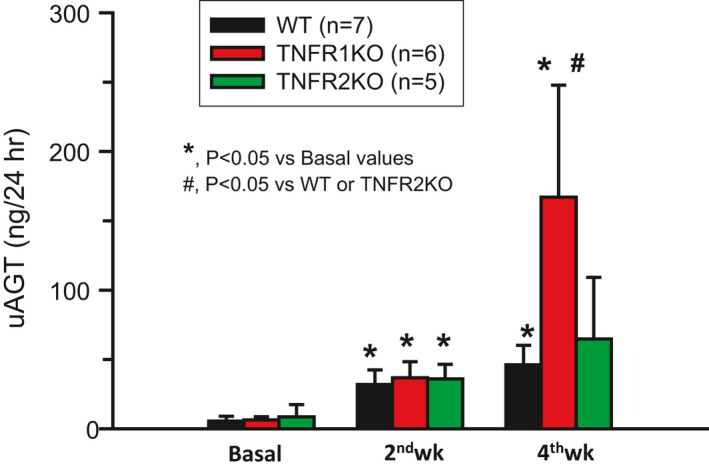
Urinary angiotensinogen excretion rate (uAGT) in wild‐type (WT) and both trains of KO mice was increased due to chronic HS (4% NaCl in diet) intake + AngII (25 ng/min) treatment for 4 weeks. However, the uAGT increases at the end 4th week were significantly higher in TNFR1KO mice than that in WT or TNFR2KO mice

Figure 2 illustrates the increases in uAGT in the three strains of mice during the chronic administration of AngII and HS intake. There were increases in uAGT by the 2nd and 4th week of the treatment period compared to the basal values in all strains of mice though the increases in TNFR2KO mice at 4th week (8 ± 7 to 65 ± 44 ng/24 h, *p *= 0.11) did not reach statistical significance level due to high variability in the results. However, the increase in uAGT by the 4th week was significantly greater (*p *< 0.05) in TNFR1KO mice (167 ± 80 ng/24 h) compared to WT mice (46 ± 16 ng/24 h) or TNFR2KO mice (65 ± 44 ng/24 h) which were not significantly different from the changes in WT mice but were less than the changes in the TNFR1KO mice.

#### 3.1.4 Glomerulosclerosis responses

**FIGURE 3:**
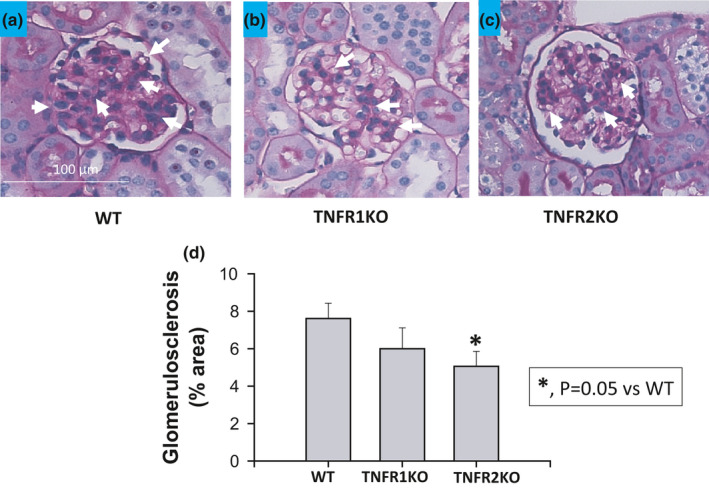
The extent of glomerulosclerosis (% area of tissue images affected by lesion) induced by chronic HS (4% NaCl in diet) intake + AngII (25 ng/min) treatment for 4 weeks was significantly attenuated in TNFR2KO (*n* = 5) but not in TNFR1KO (*n* = 6) compared to wild‐type (*n *= 7) mice. The extent of glomerulosclerosis by PAS stained images is illustrated in (a, b, and c) and the mean values of the percent area of these lesions are given in graph (d). Arrows indicating the important PAS stained images

The extent of glomerular sclerosis was evaluated by the automatic image analysis of glomeruli in PAS‐stained sections (Figure 3a–c). As shown in Figure 3d, the percent area of glomerulosclerosis was not statistically different in TNFR1KO (6.0 ± 1.1%) but significantly less in TNFR2KO mice (5.0 ± 0.8%; *p* = 0.05) than the values in WT mice (7.6 ± 0.8%). These results demonstrate that the degree of injury in TNFR2KO was reduced suggesting this receptor's involvement in the inflammatory changes leading to the development of glomerular sclerosis during chronic AngII‐treatment as observed in our previous study (Singh et al., 2013).

### 3.2 Renal interstitial collagen deposition responses

**FIGURE 4:**
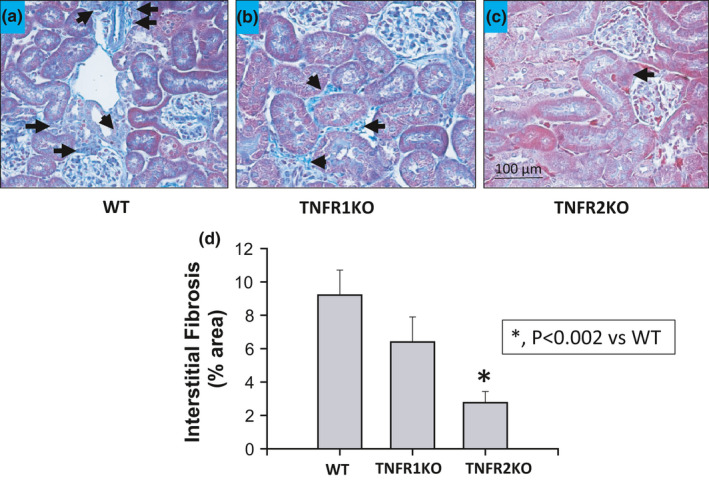
The extent of renal interstitial fibrosis (% area of tissue images affected by lesion) induced by chronic HS (4% NaCl in diet) intake + AngII (25 ng/min) treatment for 4 weeks was significantly attenuated in TNFR2KO (*n* = 5) but not in TNFR1KO (*n* = 6) compared to wild‐type (*n* = 7) mice. The extent of interstitial fibrosis by Gomori's trichrome staining images are illustrated in (a, b, and c) and the mean values of the percent area of these lesions are shown in graph (d). Arrows indicating the important trichrome stained images

Collagen deposition was revealed by the blue staining in kidney sections prepared using Gomori's trichrome staining. The extent of renal interstitial fibrosis (deposition of collagen fibers) was quantitated by the image analysis of blue staining in each microscopic field in the renal cortex areas. Collagen fibers are normally present at low levels in the renal interstitium (Figure 4a–d). Analysis using a computer‐aided semi‐automatic quantification system demonstrated that, after 4 weeks of chronic Ang II administration and HS intake, the percent area of fibrosis (collagen deposition) was similar in the renal interstitium in WT and TNFR1KO mice. Interstitial fibrosis was significantly lower (*p* < 0.05) in TNFR2KO (2.8 ± 0.7% area) but not in TNFR1KO (6.4 ± 1.5% area) mice compared with the values in WT mice (9.2 ± 1.4% area). This further indicates that the activation of TNFR2 is involved in chronic AngII‐induced interstitial collagen deposition (fibrosis) in the kidney as observed in our previous study (Singh et al., 2013).

## 4 DISCUSSION

Rodent studies have demonstrated that the chronic administration of AngII along with intake of HS‐containing diets consistently develops robust salt‐sensitive hypertension (Gonzalez‐Villalobos et al., 2008; Guzik et al., 2007; Lara et al., 2012; Singh et al., 2013; Whiting et al., 2013). In the present study, mice lacking the genes for TNF‐α receptors (TNFR1KO and TNFR2KO) also developed hypertension in response to 4 weeks of AngII treatment plus HS intake; however, it is interesting that the TNFR1KO mice showed a greater hypertensive response compared to that observed in WT mice or in TNFR2KO mice. In addition, while all groups showed increases in uAGT excretion rates, the TNFR1KO mice had greater responses in uAGT excretion rate compared to WT or TNFR2KO mice. Since uAGT provides a specific index of intrarenal AGT production in AngII‐dependent hypertension, the results suggest that the TNFR1KO mice had higher levels of intrarenal AngII. Elevated uAGT excretion rates have also been observed in patients with hypertension (Kobori et al., 2009). The uAGT excretion rate is correlated with kidney AngII content but not with plasma AngII concentrations (Kobori et al., 2003, 2007, 2009). Thus, the results of the present study indicate that TNFR1 action mitigates the hypertensive response to chronic AngII infusion with HS intake by attenuating the increase in intrarenal AGT. This present in vivo investigation reports that AngII‐induced renal AGT formation is enhanced in TNFR1KO mice indicating a protective regulatory role for TNFR1 in AGT generation. Such finding indicating a protective role for TNFR1 in AGT formation is novel and complements in vitro studies in human proximal tubular cultured cells showing that TNF‐α suppressed AGT expression via the formation of p50/p50 homodimers (Satou et al., 2010). The present study suggests that this effect was mediated via TNFR1.

An enhancement in intrarenal AGT generation is an important factor associated with the progression of AngII‐HS mediated renal injury (Gonzalez‐Villalobos et al., 2008; Lara et al., 2012; Majid, Prieto, & Navar, 2015; Navar et al., 2011). Although chronic HS intake alone does not cause changes in uAGT or in the intrarenal AGT generation, these values are markedly exacerbated when HS diets are given with AngII administration, which is also associated with greater degrees of renal injury (Lara et al., 2012). Various studies collectively indicate that chronic AngII administration augments AGT expression in proximal tubule cells leading to increases in AGT secretion into the tubular fluid and eventual increases in the uAGT excretion rate (Gonzalez‐Villalobos et al., 2008; Kobori et al., 2009; Navar et al., 2011; Satou et al., 2008). The increased intratubular AGT thus provides a substrate for increased generation of AngII along the tubular segments. Such increases in tubular AngII modulates renal tubular reabsorptive function to enhance sodium retention (Navar et al., 2011). In addition, the increase in AngII levels due to dysregulated intrarenal AGT generation also affects renal hemodynamics leading to enhanced hypertensive responses during chronic ANGII administration and HS intake (Gonzalez‐Villalobos et al., 2008; Majid, Prieto, & Navar, 2015; Navar et al., 2011).

Earlier experimental studies in rodents have provided evidence that pro‐inflammatory cytokines, particularly TNF‐α, suppress the intrarenal production of AGT that occurs in response to chronic infusion of AngII (Hao et al., 2020; Satou et al., 2010). Although AT1 receptor activation is a key factor in the stimulation of intrarenal AGT formation (Gonzalez‐Villalobos et al., 2008; Kobori et al., 2007; Navar et al., 2011), the complete pathway in the regulation of AGT formation during the condition of elevated AngII is not yet clearly understood. AngII administration is known to increase the production of proinflammatory cytokines including TNF‐α (Lara et al., 2012; Ruiz‐Ortega et al., 2002). Hypertension induced by chronic AngII administration was attenuated in mice either deficient in TNF‐α gene (Sriramula et al., 2008) or subjected to pharmacological TNF‐α blockade (Whiting et al., 2013). Although a functionally significant cross‐talk between AngII and TNF‐α in inducing hypertension has been suggested in previous studies (Urschel & Cicha, 2015; Whiting et al., 2013; Zhang et al., 2014), the exact nature of this cross‐talk has not been clearly defined, especially since it has been shown that TNF‐α administration induces natriuresis via its action on TNFR1 (Castillo et al., 2012; Majid, 2011; Shahid et al., 2008), suggesting a counter‐regulatory mechanism in hypertension via TNF‐α’s opposing action on salt retention (Majid, Prieto, & Navar, 2015; Mehaffey & Majid, 2017; Shahid et al., 2008, 2010). The results of the present study indicate that the TNF‐α activation of TNFR1 suppresses intrarenal AGT formation, thus suggesting a counter‐regulatory role of this receptor in AngII‐induced hypertension. A recent study (Hao et al., 2020) demonstrates that TNF‐α induced downregulation of AGT formation in the proximal tubules during HS intake involves the upregulation of miRNA‐133a. Although there is no direct evidence linking miRNA‐133a and TNFR1 activity in the literature yet, it has been demonstrated that TNFR1‐induced NFkB activation was suppressed by miRNA (miRNA‐218) to attenuate osteoclastogenesis in vitro indicating a possible regulation of miRNA’s in TNFR1 activity (Wang et al., 2018). Further studies will be required to identify a possible link between miRNA‐133a and TNFR1 activity in regulating renal AGT formation (Hao et al., 2020).

The present results provide further evidence that TNF‐α signaling has two distinct actions in the kidney (Mehaffey & Majid, 2017). When an increase in TNF‐α generation occurs during a hypertensive stimulus such as elevated AngII and/or increased oxidative stress, activation of TNFR1 mediates a protective function in the kidney by suppressing the increased intrarenal AGT production as well as enhancing natriuresis and thus exerts a counterregulatory role in controlling blood pressure (Mehaffey & Majid, 2017). In contrast, TNFR2 activation promotes renal tissue damage through proinflammatory pathways in such hypertensive conditions (Singh et al., 2013). In the present study, the renal injury markers, glomerulosclerosis index, and renal interstitial fibrosis, in response to HS +AngII treatment are significantly lower in magnitude in TNFR2KO but not in TNFR1KO mice compared to those in WT mice. Collectively these data indicate that the development of hypertension in response to elevated AngII and HS intake is the result of reduced TNFR1 activity that leads to an enhancement in hypertension and intrarenal AGT formation.

Mean arterial pressure changes in response to chronic AngII +HS intake in the present study were significantly higher in TNFR1KO mice compared to those in WT or in TNFR2KO mice. Similar higher MAP responses to chronic AngII administration were also reported in TNFRIKO mice compared to WT mice (Chen et al., 2010), in which a comparatively higher dose (>1.5 fold) of AngII than that used in the current study was given only for 7 days and the blood pressure was recorded during 12‐h dark periods (active period) by radio‐telemetry. In that study (Chen et al., 2010), the differences in the increases in systolic, diastolic, and mean BP in TNFR1KO mice compared to WT mice were noted statistically different from day 4 to 5 days onwards. However, such a statistical difference level between TNFR1KO and WT mice was noted in overall averages of systolic blood pressure but not in diastolic or mean blood pressure after 7 days, which could be due to reaching the maximal effects of very high dose of AngII in both the strains. However, AngII +HS intake was made for a prolonged period (4 weeks) in the current study in which MAP was significantly increased from 7th day onward.

In the present study, blood pressure in mice was recorded mostly in the mid‐day (inactive period for these nocturnal rodents) using tail‐cuff plethysmography. It should be noted here that our earlier study (Singh et al., 2013) conducted using radiotelemetry showed that the changes in daily average blood pressure in response to chronic AngII +HS intake for 2 weeks were not significantly different between TNFR1KO, TNFR2KO, and WT mice. However, further post‐analyses of data from that particular study (Singh et al., 2013) reveal that the increases in day‐time MAP were significantly higher in TNFR1KO (9 ± 1.6 mmHg; *p *< 0.05) but not in TNFR2KO (5 ± 2.2 mmHg; *p* = ns) than that in WT mice. Moreover, in separate groups of mice used for metabolic cage experiments in that study (Singh et al., 2013), it was observed that AngII +HS induced increase in day‐time systolic blood pressure (recorded by tail‐cuff method) from the basal period (0 day of the protocol) was greater in TNFR1KO mice (58 ± 6 mm) than in WT (40 ± 9 mmHg) or in TNFR2KO (48 ± 8 mmHg). Thus, these findings indicate that the lack of TNFR1 activation could have led to the non‐dipping blood pressure pattern during AngII +HS treatment. This non‐dipping pattern of blood pressure is the common feature in hypertension associated with chronic kidney diseases (Kimura et al., 2010; Ohasi et al., 2017), a condition that is characterized by an enhancement in intrarenal AGT formation (Majid, Prieto, & Navar, 2015) as well as an increase in pro‐inflammatory cytokines, particularly TNF‐α (Afsar et al., 2018; Majid, Prieto, & Navar, 2015; Rodríguez‐Iturbe et al., 2014). However, the interactive roles for TNF‐α and intrarenal AGT formation in the pathophysiology of hypertension induced by HS intake, particularly in its non‐dipping blood pressure pattern, are not yet clearly understood (Castillo et al., 2018). Thus, further comprehensive studies would be required to examine the critical role of TNF‐α and its receptor activity in this non‐dipping pattern in hypertension induced by chronic AngII treatment and HS intake.

An enhancement in intrarenal AGT generation is usually associated with the progression of hypertension and the development of renal injury (Gonzalez‐Villalobos et al., 2008; Kobori et al., 2009; Lara et al., 2012; Mehaffey & Majid, 2017). The present experiments demonstrate that the chronic HS +AngII‐induced augmentation of renal AGT formation was further enhanced in TNFR1KO, but not in TNFR2KO mice. Thus, the interactions between TNF‐α receptors and the intrarenal AGT formation may be very important to determine the degree of salt sensitivity in different individuals. Differences in the responsiveness due to the disparity of the abundance of TNFR1 and TNFR2 may help to explain the heterogeneity in blood pressure responses to HS intake in the general population.

In conclusion, the results of the present study using genetic knockout models for TNF receptors (TNFR1 and TNFR2) indicate that TNF‐α signaling has two distinct actions in the kidney. TNFR1 exerts a protective role in minimizing hypertensive response by mitigating intrarenal AGT formation, while TNFR2 contributes to renal tissue injury induced by elevated AngII level and chronic HS intake.

## CONFLICT OF INTEREST

The authors have no conflict of interest, financial or otherwise, relevant to this article.

## AUTHORS' CONTRIBUTION

DSAM conceptualized and designed the study. EM and AC performed the experiments, collected, and analyzed data with statistical analysis and prepare the figures. DSAM reviewed the processed data with statistical analysis and designed the final figures. MCP reviewed the renal histological data with PAS staining and Gomori's trichrome staining. DSAM wrote the manuscript and LGN contributed to reading and editing the manuscript as well as to review the analyzed data. All the authors reviewed the manuscript, approved the final manuscript as submitted, and agreed to be accountable for all aspects of the work.
